# Development and validation of a prognostic nomogram for predicting mortality risk in adult rheumatoid arthritis: an analysis of NHANES 1999–2018 data

**DOI:** 10.3389/fimmu.2025.1592958

**Published:** 2025-04-17

**Authors:** Xiao Chen, Haibo Gong, Jing Chen, Yuan Luo

**Affiliations:** ^1^ Department of Orthopedics, The First People’s Hospital of Neijiang, Neijiang, China; ^2^ Department of Rehabilitation, The First People’s Hospital of Neijiang, Neijiang, China; ^3^ Department of Pediatrics, The First People’s Hospital of Neijiang, Neijiang, Sichuan, China

**Keywords:** rheumatoid arthritis, mortality, inflammation, SIRI, NHANES

## Abstract

**Objective:**

This study aims to identify potential independent risk factors for rheumatoid arthritis (RA)- related mortality and develop a nomogram model to predict individualized mortality risk.

**Methods:**

This study included 310 RA patients from the National Health and Nutrition Examination Survey (NHANES) during 1999 - 2018. We applied LASSO, univariate, and multivariate logistic regression analyses to determine risk factors in the training cohort and construct a nomogram model. Calibration plots evaluated the nomogram’s accuracy. Finally, we established the nomogram’s clinical utility through DCA and performed internal validation within the training cohort.

**Results:**

Of the 310 patients, 140 experienced RA - related deaths, corresponding to a mortality rate of 45.16%. Within the training cohort, age, heart failure, and systemic inflammatory response index (SIRI) emerged as independent predictors of RA - related mortality. A nomogram model, constructed through multivariable logistic analysis, demonstrated an AUC of 0. 852 (95% CI: 0. 799 - 0. 904) in the training cohort and an AUC of 0. 904 (95% CI: 0. 846 - 0. 963) in the validation cohort. The calibration curve revealed a strong agreement between predicted and actual probabilities. In both training and validation cohorts, DCA highlighted the nomogram’s significant net benefits for predicting RA - related mortality risk.

**Conclusions:**

This study demonstrates age, heart failure, and SIRI’s ability to predict RA mortality with good discrimination and clinical utility. The model gives clinicians a simple tool to quickly identify high - risk RA patients, promoting early intervention, personalized treatment, and better prognosis.

## Introduction

Rheumatoid arthritis (RA) is a chronic inflammatory autoimmune disease that primarily targets synovial joints, resulting in joint swelling, pain, stiffness, and eventually leading to joint destruction and disability (1–3). Globally, it poses a major public health challenge, with a prevalence of approximately 0.5% to 1% (4). Future projections suggest a continued increase in the burden of RA, with an estimated 31.7 million individuals worldwide expected to be affected by 2050 (5, 6). Notably, the incidence of RA is higher in females than in males, and while it can occur at any age, the disease most commonly manifests between the ages of 30 and 60 (7, 8). Beyond causing joint disability, RA is also associated with increased long-term mortality and reduced life expectancy, with cardiovascular diseases being the primary contributor to this excess mortality (9–11). While the etiology of RA is complex and not fully elucidated, it is currently thought to involve the combined interplay of genetic and environmental factors. Genetic factors, such as specific human leukocyte antigen (HLA) genes, contribute to RA susceptibility (12–14); environmental factors, including infections, smoking, and hormonal influences, may trigger disease onset (15). A clear feature of RA is immune system dysregulation, characterized by immune cells attacking joint synovium, thereby inducing inflammation and tissue damage (16–18).

In recent years, the relationship between inflammatory markers and mortality in RA patients has garnered increasing attention. Numerous studies have shown that specific inflammatory markers, such as the neutrophil-to-lymphocyte ratio (NLR), platelet-to-lymphocyte ratio (PLR), lymphocyte-to-monocyte ratio (LMR), systemic immune-inflammation index (SII), systemic inflammatory response index (SIRI), pan-immune-inflammation value (PIV), and advanced lung cancer inflammation index (ALI), are associated with an increased risk of mortality in rheumatoid arthritis patients (10, 19–25). For instance, elevated NLR and SII are linked to higher disease activity and poorer prognosis in RA patients, potentially indicating an increased risk of cardiovascular events and mortality (22, 25). Similarly, increased SIRI and PIV have been found to be associated with adverse outcomes in RA patients, including higher mortality rates (10, 23). These inflammatory markers reflect the activation and dysregulation of the immune system in rheumatoid arthritis, the presence of chronic inflammation in the body, which promotes atherosclerosis, endothelial dysfunction, and organ damage, thereby increasing the risk of death from cardiovascular diseases and other complications.

Although increasing evidence associates inflammatory markers with mortality in RA patients, many unresolved issues and challenges remain in this field. Firstly, numerous studies have found that the above-mentioned inflammation-related indicators are associated with RA and increased mortality, but these are univariate analysis results without multivariate regression analysis, and it is not entirely clear whether they are interfered with or interacted by other factors. Secondly, the impact of confounding factors such as age, gender, and comorbidities on the relationship between inflammatory markers and mortality in RA patients is not clear. These factors may affect the levels of inflammatory markers and the risk of death, and their impact awaits further research. In addition, previous studies have not quantified the relevant indicators, making it impossible to accurately predict the probability of death in RA patients.

To address the limitations of previous studies, this study uses data from the NHANES database to investigate the association between immune-inflammatory markers and RA-related mortality. Our goal is to identify potential independent risk factors for RA-related deaths and develop a nomogram model to predict individualized mortality risk.

## Materials and methods

### Data source

The data for this study were obtained from the NHANES of the United States. This survey employed a complex, multistage stratified probability sampling method to comprehensively assess the health and nutritional status of the non-institutionalized population in the United States. The data from NHANES are nationally representative, making it an invaluable resource for conducting large-scale epidemiological studies and developing clinical prediction models. All NHANES data used in this study are publicly available at https://www.cdc.gov/nchs/nhanes. Written informed consent was obtained from all participants (or their proxies/legal guardians) for participation in the study. This study was reviewed and approved by the National Center for Health Statistics (NCHS) Ethics Review Board, with the approval numbers for each phase available at https://www.cdc.gov/nchs/nhanes/irba98.htm. All research was conducted in accordance with the Declaration of Helsinki and the Transparent Reporting of a multivariable prediction model for Individual Prognosis or Diagnosis (TRIPOD) checklist.

### Mortality linkage

The mortality data for this analysis were obtained from the NHANES public-use linked mortality files and integrated with the standard NHANES datasets using the unique respondent sequence number assigned to each participant. All-cause mortality refers to deaths from any cause, regardless of the specific underlying cause. Linkage web-page: https://www.cdc.gov/nchs/data-linkage/mortality-public.htm.

### Participant selection

**Figure 1:**
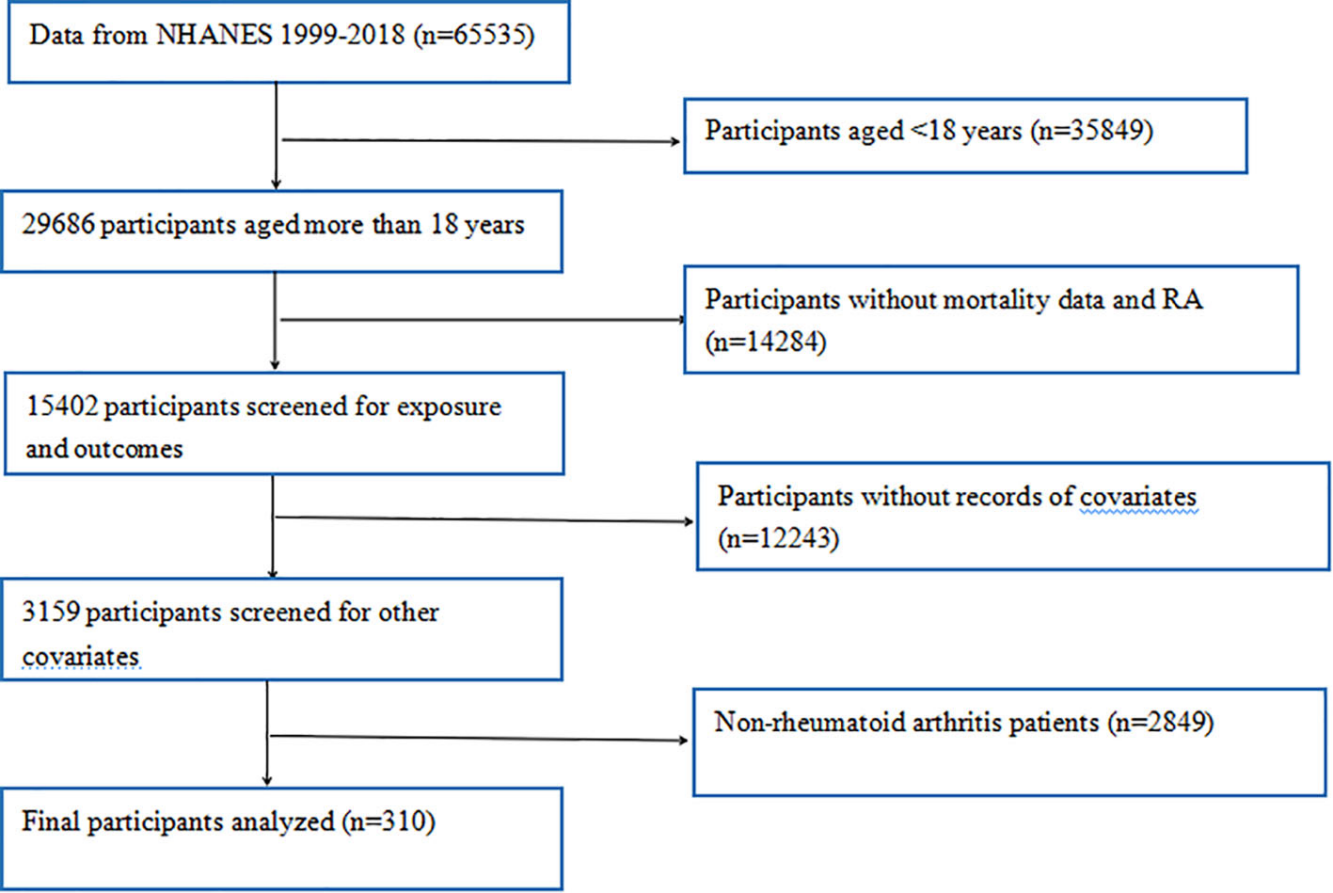
The flowchart of study participants.

Initially, 65535 participants from 10 consecutive NHANES cycles (1999 - 2018) were enrolled in this study. These participants completed extensive demographic surveys, laboratory tests, and health questionnaires during these cycles. To ensure the accuracy and reliability of our study, we conducted rigorous data screening and exclusion. First, participants aged under 18 (n = 35849) were excluded, as our study focused on adult RA (n = 29686). Subsequently, individuals lacking data on RA or mortality (n = 14284) were excluded. In addition, participants missing questionnaire and dietary data on diabetes, hypertension, smoking, alcohol consumption, heart disease, and stroke, as well as those lacking laboratory data on platelet, neutrophil, lymphocyte, and monocyte counts (n = 12243), were excluded. Moreover, non-RA patients (n = 2849) were excluded. Finally, as shown in **Figure 1**, 310 participants with RA-related deaths were included in our analysis.

### Definition of RA

The diagnosis of RA was made based on the medical condition questionnaire in the NHANES database, which asked participants the following questions. First, participants were asked, “Have you ever been told by a doctor that you had arthritis?” (MCQ160a). If the answer was “Yes,” they were then asked, “What type of arthritis?” (MCQ195; MCQ191; MCQ190), to specify the type of arthritis they had, including rheumatoid arthritis, osteoarthritis, and other types. This self - reported method of diagnosing RA has been widely used in many NHANES studies and has shown an 85% consistency between self - reported arthritis and clinical diagnosis (26, 27).

### Covariate definitions

Based on previous research, the following factors were identified as independent variables: age, sex, race, body mass index (BMI), alcohol consumption, smoking, self - reported health status, neutrophil - to - lymphocyte ratio (NLR), platelet - to - lymphocyte ratio (PLR), lymphocyte - to - monocyte ratio (LMR), systemic immune - inflammation index (SII), systemic inflammatory response index (SIRI), pan - immune - inflammation value (PIV), neutrophil - to - albumin ratio (NAR), prognostic nutritional index (PNI), systemic inflammation score (SIS), and advanced lung cancer inflammation index (ALI), etc.

Age (years) and BMI were continuous variables. Sex was divided into male and female. Race was categorized into Mexican American, other Hispanic, non - Hispanic white, non - Hispanic black, and other/multiracial. Alcohol consumption was defined by the response to the question: “Did you drink at least 12 drinks of any type of alcohol in any year?”, and was grouped into two categories (yes or no). Smoking status was divided into smoker and never smoker based on the response to the question “Have you smoked at least 100 cigarettes in your life?”. In our study, hypertension was defined as self - reported physician - diagnosed, use of antihypertensive medication, or blood pressure≥140/90 mmHg. Diabetes mellitus (DM) status was defined as follows: Diabetes” (physician self - reported diagnosis, HbA1c≥6.5%, fasting plasma glucose (FPG)≥7.0 mmol/L, random glucose≥11.1 mmol/L, 2 - hour glucose tolerance test≥11.1 mmol/L, or use of diabetes medication or insulin). Blood biomarkers included albumin (ALB), neutrophil count (NC), lymphocyte count (LC), monocyte count (MC), and platelet count (PLT). As previously described, NLR was calculated as NC/LC; PLR was calculated as PLT/LC; LMR was calculated as LC/MC; SII was calculated as (PLT*NC)/LC; SIRI was calculated as (NC*MC)/LC; PIV was calculated as (NC*PLT*MC)/LC; NAR was calculated as NC/ALB; ALI was calculated as (BMI*Alb)/NLR; PNI was calculated as ALB + 5*LC; SIS was calculated based on LMR and ALB, with 0 points for LMR≥2.17 and ALB≥39.8 g/L, 1 point for LMR < 2.17 or ALB < 39.8 g/L, and 2 points for LMR < 2.17 and ALB < 39.8 g/L. Potential confounding variables associated with RA mortality were selected from published RA studies, including demographics (age, gender, race, education), behavioral factors (smoking, alcohol use), clinical characteristics (cardiovascular diseases, BMI), and inflammation markers.

### Statistical analysis

NHANES employs a complex multistage sampling design. Sample - weighted data requires calculation based on this design. However, for machine learning model development, we used unweighted data from the NHANES database. Unlike weighted data, which estimates national incidence and prevalence, our focus was exploring the relationship between RA - related mortality and individual characteristics to build a model.

Data statistical analysis was conducted in R (version 4.2.2). Continuous variables are expressed as mean ± SD, with inter - group differences assessed by t - tests. Categorical variables are presented as frequencies and percentages, compared using chi - square tests. All tests were two - sided, with p < 0.05 indicating statistical significance.

We randomly split 310 participants into training (217) and validation (93) cohorts at a 7:3 ratio. The training cohort served for model development, while the validation cohort was for internal validation. To identify predictors of RA - related mortality and reduce variable collinearity, we applied Least Absolute Shrinkage and Selection Operator (LASSO) logistic regression. This technique minimizes coefficients of less influential variables to zero, enhancing model prediction accuracy. We evaluated and optimized the LASSO model via cross - validation, dividing the dataset into ten subsets. The model underwent iterative training and assessment on these subsets to determine effectiveness and optimal parameters. During cross - validation, we typically generated a model performance plot for different lambda values to assess effectiveness across settings. “Minimum deviation” refers to the lambda value yielding the smallest deviation via cross - validation, signifying the best data fit. We selected this lambda value as optimal. LASSO logistic regression in the training set identified independent predictors, forming an RA - related mortality prediction model. We then established a clinical diagnostic nomogram using feature variables.

Model performance assessment involved three metrics: Area under the Curve (AUC), calibration curves, and decision curve analysis (DCA). AUC reflects a model’s discrimination ability between patients and healthy individuals; higher values mean better diagnostic accuracy. Calibration curves illustrate the predicted - observed outcome relationship. Using 1000 bootstrap samples to plot these curves enhanced precision and reduced over-fitting bias. The closer the calibration curve to the ideal, the higher the model’s prediction accuracy. DCA evaluates a model’s clinical utility by analyzing clinical net benefit across different threshold probabilities, providing a comprehensive and clinically relevant performance assessment. We also conducted Kaplan - Meier analysis based on key death - risk - related variables to clarify underlying relationships.

## Results

### Baseline characteristics of participants

After stepwise screening, this study included 310 participants from the NHANES database (1999 - 2018) meeting the inclusion and exclusion criteria for analysis. Among them, 140 experienced RA - related deaths (140/310), with a mortality rate of 45.16%. These participants were randomly divided into training (217) and validation (93) sets at a 7:3 ratio. The training set had a mean age of 67 ± 13 years, with 95 RA - related deaths (43.78%). The validation set had a mean age of 69 ± 12 years, with 45 RA - related deaths (48.39%). In this study, we analyzed the baseline demographic and clinical characteristics of the participants across the different cohorts. Apart from RAR (P = 0.047), no significant differences were found between the two groups in other indicators (P > 0.05), and these had no significant impact on the outcome, indicating a comparable baseline for predictive research (**Table 1**).

**Table 1:** **|** Patient demographics and baseline characteristics.

Characteristic	Cohort		P-value
	Training Cohort, N = 217	Internal Test Cohort, N = 93	
Gender, n(%)			0.949
Male	39 (18.0%)	17 (18.3%)	
Female	178 (82.0%)	76 (81.7%)	
Age(years)			0.130
Mean ± SD	67 ± 13	69 ± 12	
Race, n(%)			0.065
Mexican American	26 (12.0%)	5 (5.4%)	
Other Hispanic	20 (9.2%)	3 (3.2%)	
Non-Hispanic White	132 (60.8%)	61 (65.6%)	
Non-Hispanic Black	33 (15.2%)	19 (20.4%)	
Other Race	6 (2.8%)	5 (5.4%)	
BMI			0.897
Mean ± SD	31 ± 7	31 ± 9	
Hypertension, n(%)			0.348
Yes	165 (76.0%)	66 (71.0%)	
No	52 (24.0%)	27 (29.0%)	
Diabetes, n(%)			0.974
Yes	40 (18.4%)	17 (18.3%)	
No	177 (81.6%)	76 (81.7%)	
Heart failure, n(%)			0.929
Yes	18 (8.3%)	8 (8.6%)	
No	199 (91.7%)	85 (91.4%)	
Coronary heart disease, n(%)			0.209
Yes	18 (8.3%)	12 (12.9%)	
No	199 (91.7%)	81 (87.1%)	
Angina, n(%)			0.213
Yes	20 (9.2%)	13 (14.0%)	
No	197 (90.8%)	80 (86.0%)	
Heart attack, n(%)			0.926
Yes	17 (7.8%)	7 (7.5%)	
No	200 (92.2%)	86 (92.5%)	
Stroke, n(%)			0.847
Yes	15 (6.9%)	7 (7.5%)	
No	202 (93.1%)	86 (92.5%)	
Smoking, n(%)			0.033
Yes	64 (29.5%)	39 (41.9%)	
No	153 (70.5%)	54 (58.1%)	
Drinking, n(%)			0.306
Yes	117 (53.9%)	56 (60.2%)	
No	100 (46.1%)	37 (39.8%)	
NLR			0.828
Mean ± SD	2.30 ± 1.43	2.34 ± 1.12	
PLR			0.262
Mean ± SD	138 ± 55	145 ± 55	
LMR			0.760
Mean ± SD	4.00 ± 1.57	4.07 ± 1.87	
RAR			0.047
Mean ± SD	0.31 ± 0.04	0.33 ± 0.06	
SII			0.914
Mean ± SD	594 ± 384	599 ± 321	
SIRI			0.545
Mean ± SD	1.28 ± 0.83	1.22 ± 0.68	
PIV			0.474
Mean ± SD	334 ± 244	314 ± 208	
NAR			0.397
Mean ± SD	0.10 ± 0.04	0.10 ± 0.03	
PNI			0.054
Mean ± SD	52.1 ± 4.8	50.9 ± 5.2	
SIS			0.315
Mean ± SD	0.35 ± 0.53	0.42 ± 0.61	
ALI			0.517
Mean ± SD	694 ± 360	664 ± 392	
Follow-up(month)			0.966
Mean ± SD	136 ± 55	135 ± 48	

BMI, body mass index; NLR, neutrophil-to-lymphocyte ratio; PLR, platelet-to-lymphocyte ratio; LMR, lymphocyte-to-monocyte ratio; SII, systemic immune-inflammatory index; SIRI, systemic inflammatory response index; PIV, pan-immune-inflammation value; NAR, neutrophil-to-albumin ratio; PNI, Prognostic Nutritional Index; SIS, systemic inflammation score; ALI, advanced lung cancer inflammation index.

In the training set, the Wilcoxon test or chi - square test was employed to compare various indicators between the death group and the non - death group. The results revealed statistically significant differences in age (P < 0.001), race (P = 0.003), BMI (P < 0.001), heart failure (P = 0.002), NLR (P < 0.001), PLR (P = 0.005), LMR (P < 0.001), SII (P = 0.006), SIRI (P < 0.001), PIV (P < 0.001), PNI (P < 0.001), SIS (P < 0.001), and ALI (P < 0.001) (**Table 2**).

**Table 2:** Comparison of variables between death group and non-death group.

Characteristics	Training Cohort			Internal Test Cohort		
	Non-death group N = 122	Death group N = 95	P-value	Non-death group N = 48	Death group N = 45	P-value
Gender, n(%)			0.492			0.136
Male	20 (16%)	19 (20%)		6 (13%)	11 (24%)	
Female	102 (84%)	76 (80%)		42 (88%)	34 (76%)	
Age(years)			<0.001			<0.001
Mean ± SD	62 ± 12	73 ± 11		62 ± 12	77 ± 8	
Race, n(%)			0.003			0.082
Mexican American	18 (15%)	8 (8%)		4 (8%)	1 (2%)	
Other Hispanic	17 (14%)	3 (3%)		2 (4%)	1 (2%)	
Non-Hispanic White	62 (51%)	70 (74%)		27 (56%)	34 (76%)	
Non-Hispanic Black	20 (16%)	13 (14%)		10 (21%)	9 (20%)	
Other Race	5 (4%)	1 (1%)		5 (10%)	0 (0%)	
BMI			<0.001			0.295
Mean ± SD	32 ± 7	29 ± 7		32 ± 8	30 ± 10	
Hypertension, n(%)			0.227			0.006
Yes	89 (73%)	76 (80%)		28 (58%)	38 (84%)	
No	33 (27%)	19 (20%)		20 (42%)	7 (16%)	
Diabetes, n(%)			0.215			0.341
Yes	26 (21%)	14 (15%)		7 (15%)	10 (22%)	
No	96 (79%)	81 (85%)		41 (85%)	35 (78%)	
Heart failure, n(%)			0.002			0.027
Yes	4 (3%)	14 (15%)		1 (2%)	7 (16%)	
No	118 (97%)	81 (85%)		47 (98%)	38 (84%)	
Coronary heart disease, n(%)			0.579			0.905
Yes	9 (7%)	9 (9%)		6 (13%)	6 (13%)	
No	113 (93%)	86 (91%)		42 (88%)	39 (87%)	
Angina, n(%)			0.125			0.306
Yes	8 (7%)	12 (13%)		5 (10%)	8 (18%)	
No	114 (93%)	83 (87%)		43 (90%)	37 (82%)	
Heart attack, n(%)			0.193			0.709
Yes	7 (6%)	10 (11%)		3 (6%)	4 (9%)	
No	115 (94%)	85 (89%)		45 (94%)	41 (91%)	
Stroke, n(%)			0.189			0.005
Yes	6 (5%)	9 (9%)		0 (0%)	7 (16%)	
No	116 (95%)	86 (91%)		48 (100%)	38 (84%)	
Smoking, n(%)			0.760			0.371
Yes	37 (30%)	27 (28%)		18 (38%)	21 (47%)	
No	85 (70%)	68 (72%)		30 (63%)	24 (53%)	
Drinking, n(%)			0.247			0.702
Yes	70 (57%)	47 (49%)		28 (58%)	28 (62%)	
No	52 (43%)	48 (51%)		20 (42%)	17 (38%)	
NLR			<0.001			0.004
Mean ± SD	1.94 ± 0.92	2.77 ± 1.80		2.01 ± 0.97	2.68 ± 1.17	
PLR			0.005			0.066
Mean ± SD	128 ± 54	149 ± 54		135 ± 53	156 ± 55	
LMR			<0.001			<0.001
Mean ± SD	4.51 ± 1.34	3.36 ± 1.60		4.82 ± 1.94	3.27 ± 1.42	
RAR			0.227			0.878
Mean ± SD	0.31 ± 0.04	0.32 ± 0.04		0.33 ± 0.08	0.33 ± 0.04	
SII			0.006			0.059
Mean ± SD	531 ± 356	676 ± 404		538 ± 323	663 ± 309	
SIRI			<0.001			<0.001
Mean ± SD	0.99 ± 0.51	1.64 ± 1.01		0.93 ± 0.45	1.54 ± 0.75	
PIV			<0.001			0.002
Mean ± SD	275 ± 186	410 ± 287		249 ± 149	384 ± 239	
NAR			0.050			0.265
Mean ± SD	0.10 ± 0.04	0.11 ± 0.05		0.096 ± 0.036	0.104 ± 0.034	
PNI			<0.001			0.055
Mean ± SD	53.1 ± 4.5	50.7 ± 4.8		51.9 ± 5.9	49.8 ± 4.2	
SIS			<0.001			0.299
Mean ± SD	0.22 ± 0.42	0.51 ± 0.62		0.35 ± 0.48	0.49 ± 0.73	
ALI			<0.001			<0.001
Mean ± SD	807 ± 326	550 ± 351		793 ± 462	526 ± 236	

BMI, body mass index; NLR, neutrophil-to-lymphocyte ratio; PLR, platelet-to-lymphocyte ratio; LMR, lymphocyte-to-monocyte ratio; SII, systemic immune-inflammatory index; SIRI, systemic inflammatory response index; PIV, pan-immune-inflammation value; NAR, neutrophil-to-albumin ratio; PNI, Prognostic Nutritional Index; SIS, systemic inflammation score; ALI, advanced lung cancer inflammation index.

### Selection of main predictors of RA-related mortality

**Figure 2:**
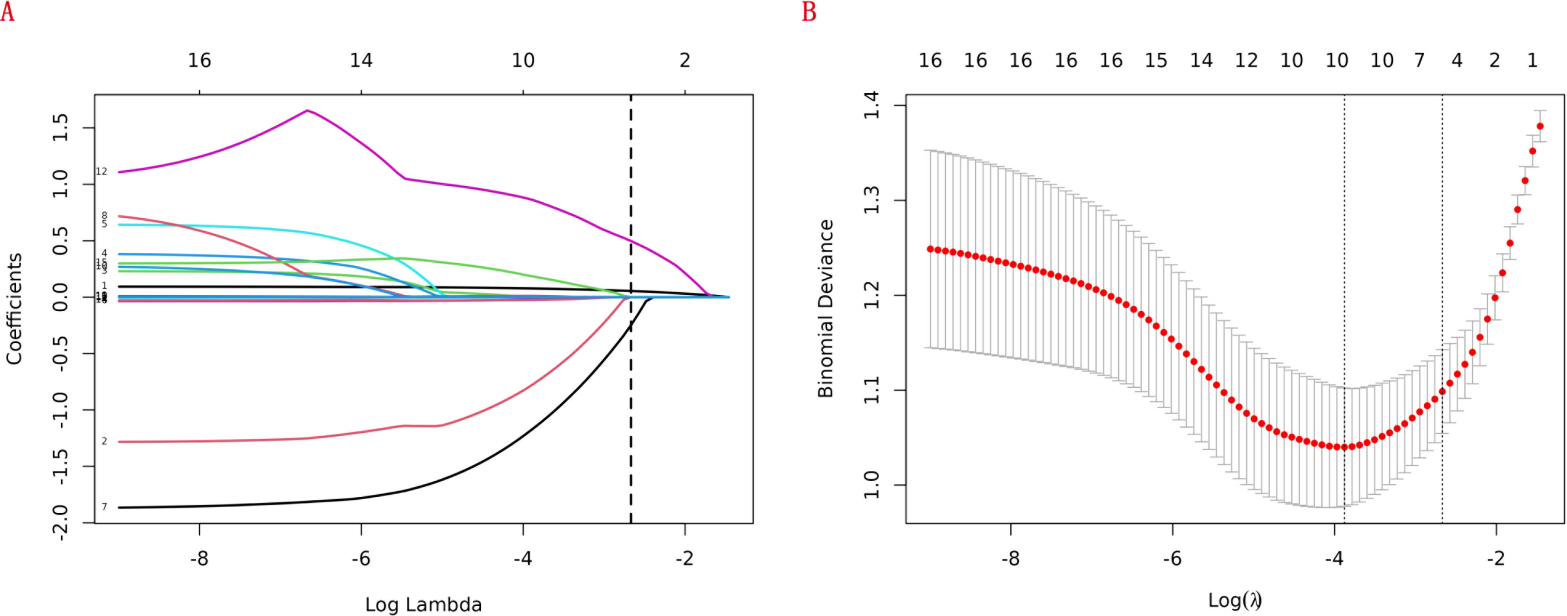
Lasso regression cross-validation plot **(A)** and lasso regression coefficient path plot **(B)**.

**Figure 3:**
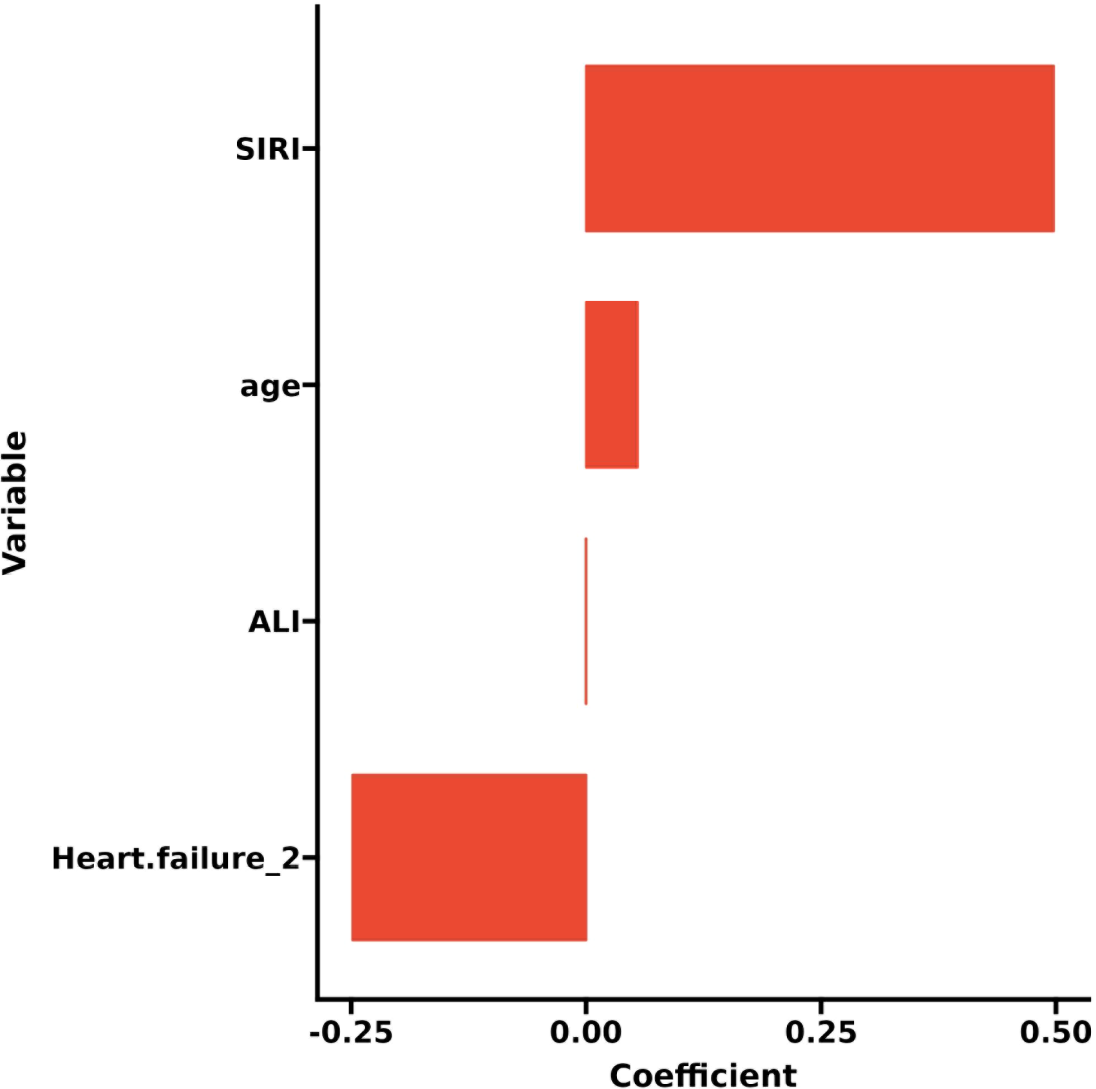
Plot of coefficient profile.

LASSO regression adds an L1 regularization term (absolute value penalty) to ordinary least squares regression. It selectively shrinks some coefficients to zero, identifying the most critical features. This technique reduces over-fitting, enhances generalization, and promotes feature selection, particularly for correlated features, improving model interpretability and performance. Coefficient shrinkage in LASSO regression is achieved by minimizing a loss function that includes the L1 regularization term. This process sets some coefficients to zero, effectively eliminating non - essential features. Using LASSO regression, we selected 4 significant predictors from the 24 feature variables in the training cohort: age, heart failure, SIRI and ALI (**Figures 2A, B**). The coefficients are shown in **Table 3**, and a coefficient profile is plotted in **Figure 3**.

**Table 3:** The coefficients of Lasso regression analysis.

variable	Coefficient
(Intercept)	-4.1601061499
age	0.0547509970
Race_Other Hispanic	0.0000000000
Race_Non-Hispanic White	0.0000000000
Race_Non-Hispanic Black	0.0000000000
Race_Other Race	0.0000000000
BMI	0.0000000000
Heart.failure_No	-0.2485664004
NLR	0.0000000000
PLR	0.0000000000
LMR	0.0000000000
SII	0.0000000000
SIRI	0.4975465642
PIV	0.0000000000
PNI	0.0000000000
SIS	0.0000000000
ALI	-0.0002682872

BMI, body mass index; NLR, neutrophil-to-lymphocyte ratio; PLR, platelet-to-lymphocyte ratio; LMR, lymphocyte-to-monocyte ratio; SII, systemic immune-inflammatory index; SIRI, systemic inflammatory response index; PIV, pan-immune-inflammation value; NAR, neutrophil-to-albumin ratio; PNI, Prognostic Nutritional Index; SIS, systemic inflammation score; ALI, advanced lung cancer inflammation index.

### Construction of a new prediction model of RA-related mortality

**Figure 4:**
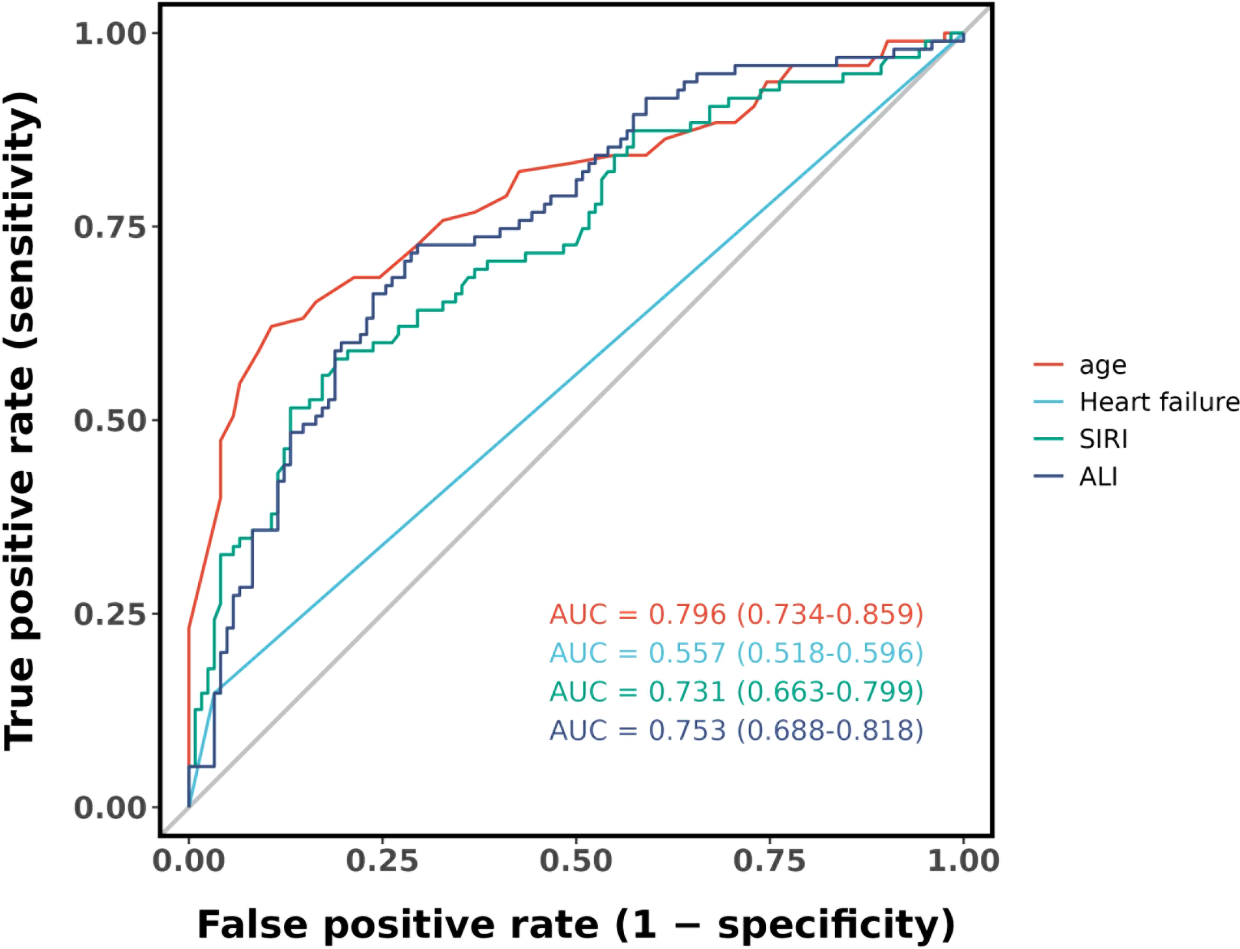
ROC curve analysis 4 candidate diagnostic indicators.

**Figure 5:**
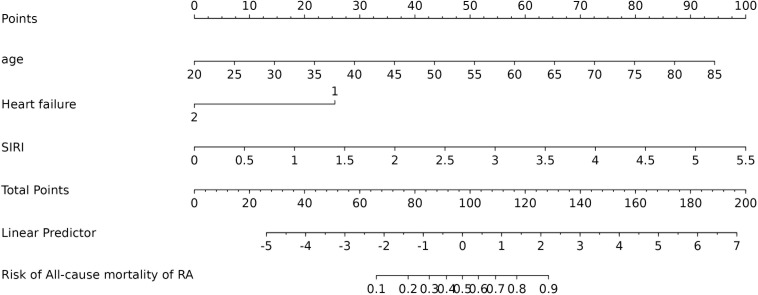
Nomogram of probability to develop RA - related mortality using age, heart failure, and SIRI. To use the nomogram, draw an upward vertical line from each covariate to the points bar to calculate the number of points. Based on the sum of the covariate points, draw a downward vertical line from the total points line to calculate the probability of developing RA - related mortality.

**Figure 4** shows that the AUC of the above variables all exceed 0.5, among which the AUC of age is 0.796, heart failure is 0.557, SIRI is 0.731, and ALI is 0.753. Next, to establish a new predictive model, we performed multivariable logistic analysis on the training cohort, eliminating the not significant indicator ALI (P = 0.341) (**Table 4**). The final model includes 3 independent predictors (age, heart failure, SIRI). These mutually independent predictors were combined into a nomogram to quantify the probability of RA - related mortality (**Figure 5**). For example, in this study, for a 79 - year - old female patient with heart failure, the “age” score was 85, “heart failure” was 25, and SIRI was 25 (SIRI = 1.4412). Therefore, if the total score of the three predictors in the histogram is 135, the corresponding predicted probability is 0.9, indicating a 90% mortality risk for the RA patient.

**Table 4:** Results of multivariate logistic regression for training cohort.

Characteristic	N	Event N	OR	95% CI	P-value
Age	217	95	1.10	1.07, 1.14	<0.001
Heart failure					
Yes	18	14	ref	ref	
No	199	81	0.15	0.04, 0.67	0.012
SIRI	217	95	2.86	1.40, 5.85	0.004
ALI	217	95	1.00	1.00, 1.00	0.341

SIRI, systemic inflammatory response index; ALI, advanced lung cancer inflammation index.

### Kaplan–Meier survival analysis of the 3 independent predictive factors

**Figure 6:**
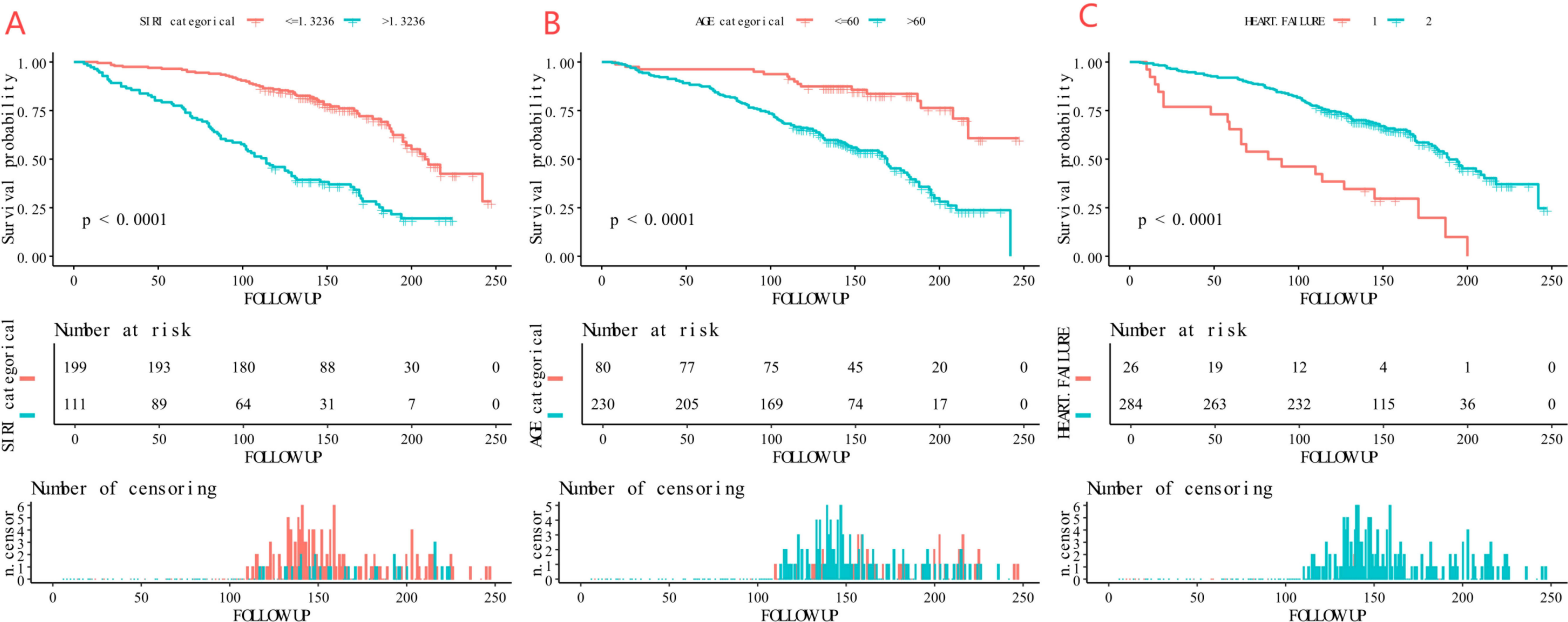
Kaplan-Meier curves of the survival rate of participants with age, heart failure, and SIRI (**A**: age, **B**: heart failure, **C**: SIRI).

Kaplan-Meier survival analysis showed that high-risk patients - older age (>60 years), with heart failure, and high SIRI value (>1.3236) - had significantly higher long-term mortality than low-risk patients (all P-values < 0.001) (**Figure 6**).

### Performance of the new nomogram of RA-related mortality in AUC, and calibration curve

**Figure 7:**
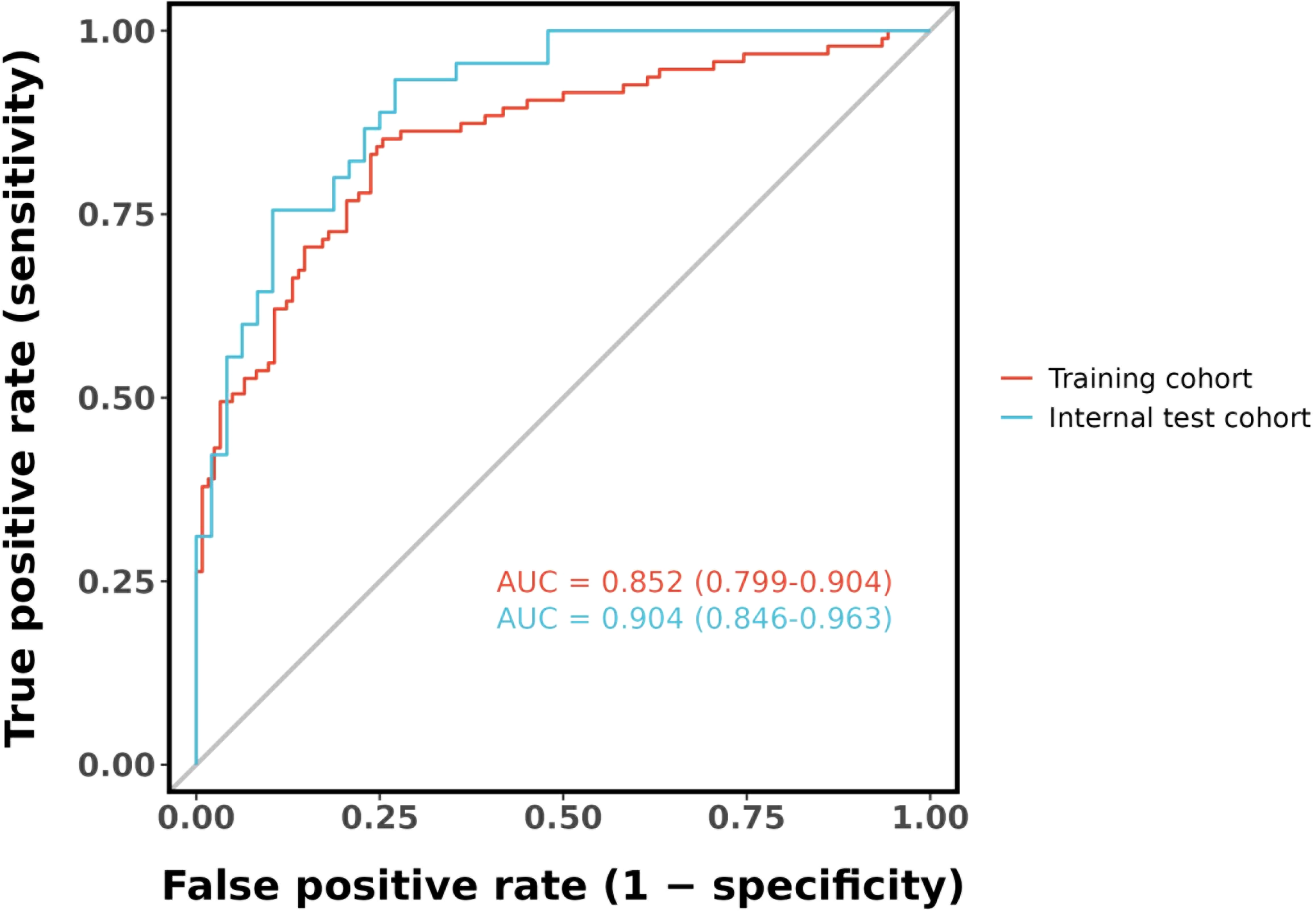
ROC curve for the nomogram based on the training cohort (The AUC is 0.852) and internal validation cohort (The AUC is 0.904).

**Figure 8:**
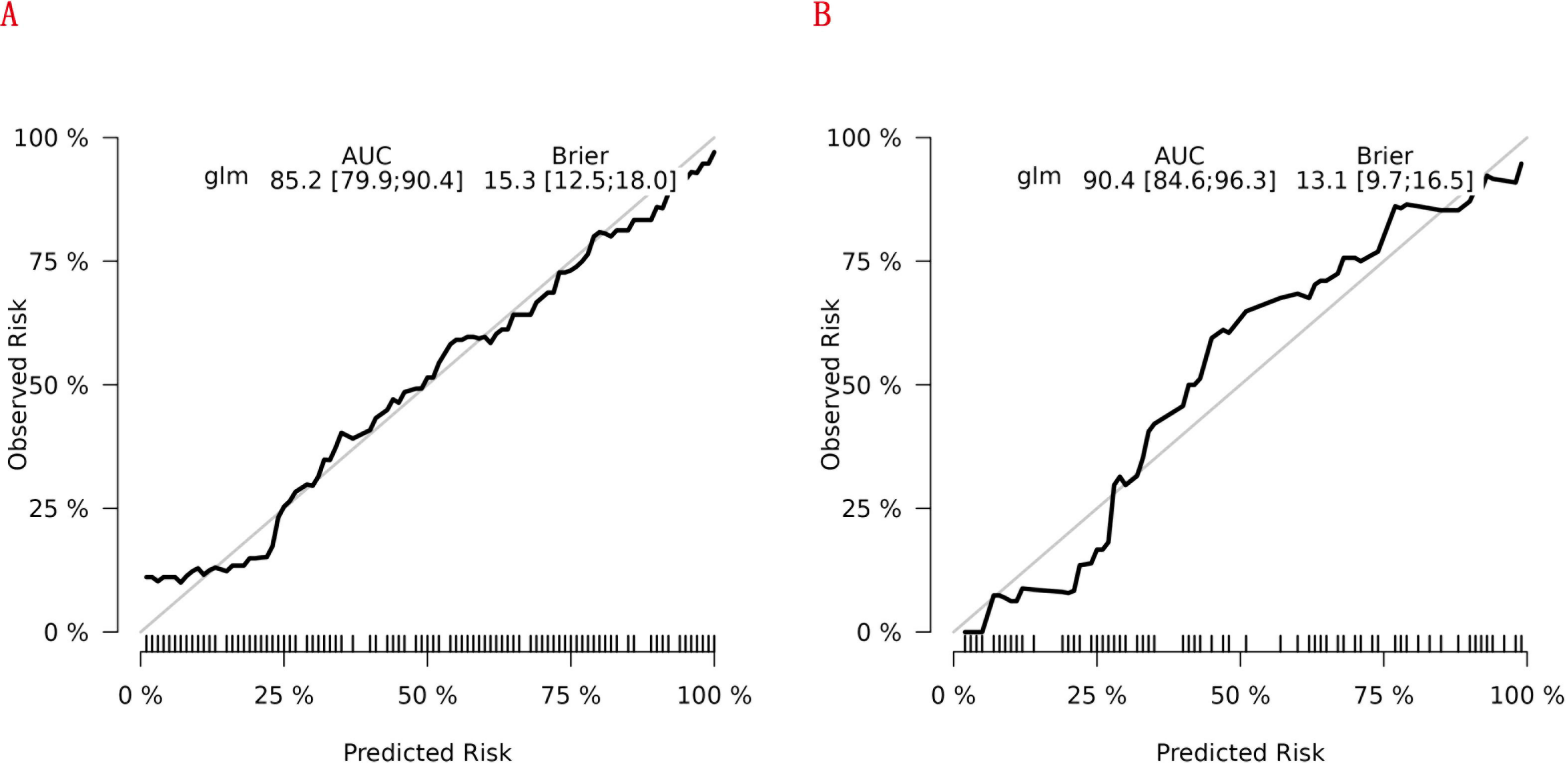
Calibration curves of the nomogram for predicting RA - related mortality from the training cohort **(A)** and the internal validation cohort **(B)**.

**Figure 7** shows the model’s performance with an AUC of 0.852 (95% CI 0.799 - 0.904) in the training cohort and an AUC of 0.904 (95% CI 0.846 - 0.963) in the internal validation cohort. Both AUC values exceed those of single indicators, indicating good predictive ability. The calibration plots for both cohorts (**Figures 8A, B**) show well - aligned calibration curves with the ideal curve, demonstrating strong consistency between predicted and observed outcomes. These results highlight the nomogram’s good predictive value and discrimination.

### Evaluation of clinical utility of the new nomogram of RA-related mortality

**Figure 9:**
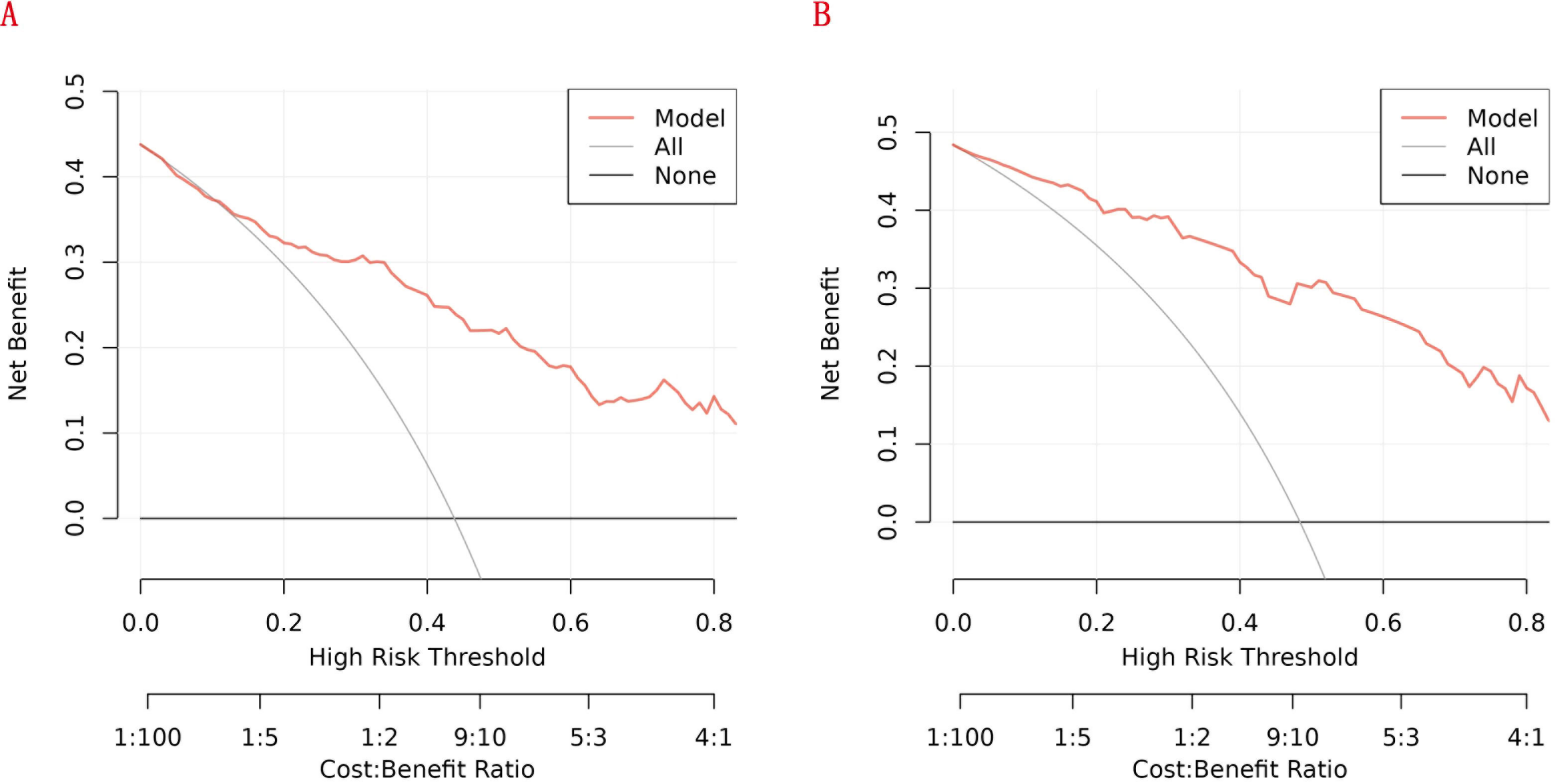
Decision curve analysis **(DCA)** of the nomogram: **(A)** the training cohort; **(B)** the internal validation cohort.

We further conducted DCA to evaluate the clinical utility of the newly developed RA - related mortality nomogram. In **Figures 9A,B**, the nomogram exhibited notable net benefits across both the training and validation cohorts. These findings suggest that the newly established RA - related mortality nomogram holds significant clinical practical value.

## Discussion

Although the exact etiology and pathogenesis of RA remain unclear, RA is evidently an autoimmune disease characterized by immune dysregulation. Immune cells attack host tissues, inducing inflammation and tissue damage (28, 29). For example, immune attacks on blood vessels can lead to vasculitis, while those on lung tissue can result in pulmonary interstitial fibrosis and rheumatoid nodule formation. These nodules may liquefy and be coughed out, causing lung cavitation. This impairs respiratory function and can lead to respiratory failure in severe cases (30–33). When RA affects the heart, it can cause myocarditis, pericarditis, and myocardial ischemia, which can be life - threatening (34–37). Thus, there is a growing need for a simple and user - friendly RA - related mortality prediction model to identify individuals at risk. A nomogram is a visual predictive tool that helps screen for potentially diseased individuals and has been widely used in many fields. In our study, we developed a model based on the NHANES database (1999 - 2018) for RA - related deaths. By examining these characteristic variables, we identified three optimal predictors (age, heart failure, and SIRI) and constructed an effective RA - related mortality probability prediction nomogram. The risk probability of RA - related death can be estimated by calculating scores. The model has high predictive performance (training cohort AUC 0.852, validation cohort AUC 0.904), strong discrimination, and clinical applicability. It helps primary care physicians quickly and accurately assesses RA - related mortality risk. Similarly, our findings support this view that the Kaplan–Meier analysis demonstrated a notably reduced survival probability for subjects exhibiting increased SIRI values. Our model simplifies medical procedures and promotes early detection and treatment.

In RA pathogenesis and progression, inflammatory cells and mediators are pivotal (38). Immune cells (neutrophils, lymphocytes, monocytes) interact and release various inflammatory mediators, driving the inflammatory response. Recently, many studies focused on inflammatory markers (NLR, PLR, LMR, SII, PIV, and SIRI) application value in RA. They reflect pro - inflammatory and anti - inflammatory immune response balance, offering RA disease activity and prognosis assessment clues (39).

Among them, NLR, reflecting the body’s inflammatory state and immune function, correlates closely with increased cardiovascular mortality in RA patients. This may stem from neutrophils releasing neutrophil extracellular traps (NETs) in RA, driving pathological processes like synovitis, cartilage destruction, and bone erosion. NETs can also promote atherosclerosis and endothelial damage, thus raising cardiovascular event risks (40, 41). Zhou et al. (2023) demonstrated NLR as a key predictor of cardiovascular and all - cause mortality in RA patients (27). PLR indicates platelet activation. Platelets, crucial in blood coagulation, worsen inflammation via interactions with white blood cells and endothelial cells (42). In RA patients, high PLR suggests reduced lymphocytes, hinting at impaired anti - inflammatory immunity, which exacerbates systemic inflammation and boosts thrombosis and cardiovascular event risks (43–45). Similarly, Liu et al. (2023) reported SII’s prognostic value in RA - related outcomes (26). SII, considering neutrophil, platelet, and lymphocyte counts, links to RA disease activity and mortality. It better reflects the body’s inflammatory and thrombotic risks, enriching RA clinical management information (23–25).

As a comprehensive marker of the body’s inflammatory state, SIRI integrates neutrophils, monocytes, and lymphocytes, offering a more nuanced assessment of systemic inflammation. In RA patients, elevated SIRI often indicates increased inflammatory activity and severe immune imbalance (46, 47). Neutrophils play a crucial role in early inflammation, releasing substances like NETs that directly damage joint synovium, promote oxidative stress, and increase cardiovascular disease risk (40, 48). Monocytes differentiate into macrophages and osteoclasts, driving synovial hyperplasia, joint inflammation, and bone destruction (49–51). A relative decrease in lymphocytes may reflect impaired immune regulation, making it difficult to control inflammation (52–54). Specifically, elevated SIRI impacts RA patients in several ways: (1) Increased cardiovascular disease risk: RA patients already face a higher risk of cardiovascular disease (55), which is further aggravated by elevated SIRI (46, 56). Persistent pro-inflammatory cytokines like TNF-α and IL-6 damage vascular endothelial cells, resulting in endothelial dysfunction. This dysfunction increases the expression of adhesion molecules on the endothelial surface, promoting platelet aggregation and the adhesion and infiltration of inflammatory cells, accelerating atherosclerotic plaque formation (57, 58). RA patients often demonstrate the “lipid paradox,” where despite lower levels of total cholesterol, LDL, and HDL, cardiovascular risk increases (59–61). Inflammation alters HDL particle subcomponents and structure, reducing its anti-atherosclerotic function and increasing LDL-C oxidation, further accelerating plaque formation and promoting plaque instability. Neutrophils and monocytes interact in the inflammatory response. Neutrophils release NETs, damaging joint synovium and increasing cardiovascular disease risk (40, 48, 62), while monocytes differentiate into macrophages that secrete inflammatory mediators, worsening vascular inflammation (51). (2)Promotion of respiratory complications: RA can impact the respiratory system, causing complications like interstitial lung disease, pulmonary fibrosis, and rheumatoid nodules (63). Patients with elevated SIRI exhibit more active systemic inflammation, with immune cell infiltration and activation in lung tissue exacerbating pulmonary inflammation and fibrosis (30, 32, 64–66). For example, neutrophil-released elastase and myeloperoxidase damage alveolar epithelial cells and lung interstitium, while monocyte-derived macrophages secrete inflammatory mediators, stimulating fibroblast proliferation and collagen synthesis (67). These pathological changes result in declining lung function, symptoms like dyspnea and cough, and potentially respiratory failure and increased mortality risk. (3) Exacerbation of immune system imbalance: Elevated SIRI indicates immune system imbalance in RA patients, characterized by an imbalance between pro-inflammatory and regulatory T cells. Pro-inflammatory T cells like Th1 and Th17 are overactivated, secreting large amounts of pro-inflammatory cytokines that drive inflammation (68). In contrast, decreased numbers and function of regulatory T cells (Tregs) prevent effective suppression of excessive inflammation, resulting in persistent autoimmune attacks (69–71). This immune system imbalance not only worsens joint inflammation and damage but also makes other organs and tissues more susceptible to immune-mediated injury. For example, immune attacks on vascular endothelial cells can cause vasculitis, affecting blood supply to multiple organs, while attacks on heart tissue can lead to myocarditis and pericarditis, impairing heart function (72, 73). (4) Systemic inflammation damage to organs: The systemic inflammation of RA damages various organs through multiple pathways, increasing mortality risk. In the cardiovascular system, in addition to atherosclerosis and endothelial dysfunction, inflammation can directly damage myocardial cells and cause myocardial fibrosis, impairing cardiac contraction and relaxation (72, 73). In the kidneys, chronic inflammation can lead to glomerulonephritis and interstitial nephritis, impairing kidney function and causing metabolic waste and fluid retention, exacerbating systemic inflammation (74, 75). Additionally, inflammation can impact the endocrine system, such as disrupting insulin signaling, leading to insulin resistance and abnormal glucose metabolism (75), further increasing the risk of cardiovascular disease and other complications. Previous research by Yin based on the NHANES database demonstrated a significant association between elevated SIRI and increased all-cause and cardiovascular mortality in RA patients (76). In RA patients, elevated SIRI not only indicates increased disease activity but also predicts higher mortality risk. Similarly, our study demonstrated that SIRI is strongly associated with increased mortality and is an independent risk factor for increased death risk in RA patients.

Our study demonstrated that age and heart failure are independent risk factors for rheumatoid arthritis (RA) - related mortality. Aging is associated with immunosenescence, characterized by a decline in adaptive immunity and a transition to a pro - inflammatory state, often termed “inflammaging” (77, 78). This chronic low - grade inflammation aggravates RA disease activity and heightens the risk of comorbidities such as cardiovascular diseases. Heart failure, a common RA complication, further elevates mortality risk through shared inflammatory pathways. RA - related inflammation contributes to myocardial fibrosis, endothelial dysfunction, and atherosclerosis, resulting in systolic and diastolic heart failure (79–81). Elevated pro - inflammatory cytokines like TNF - α and IL - 6 among RA patients have been linked to heart failure pathogenesis, emphasizing the interaction between systemic inflammation and cardiovascular dysfunction. In summary, age and heart failure contribute to RA - related mortality through mechanisms involving chronic inflammation, immune dysregulation, and cardiovascular dysfunction (7, 82, 83).

We propose the following recommendations for applying this model to clinical practice and integrating it with other clinical indicators for comprehensive assessment: 1. Risk Stratification and Triage Management: “A scoring system based on age, heart failure status, and SIRI enables clinicians to rapidly identify RA patients needing priority intervention. For example, high - risk patients (elderly, with heart failure, and SIRI>1.326) should be referred to a joint clinic of rheumatology and cardiology for intensified anti - inflammatory therapy (e.g., biologics) and cardiovascular risk management (e.g., statins).”. 2. Dynamic Monitoring and Treatment Adjustment: “We suggest moderate - to - high - risk patients undergo rechecks of SIRI and cardiac function indicators such as BNP and echocardiography every six months. This dynamic risk assessment helps monitor disease progression. If SIRI continues to rise or new heart failure symptoms emerge, the treatment plan should be adjusted, for instance, by upgrading anti - inflammatory drugs or incorporating heart failure - targeted therapies.”. 3. Integration with Other Clinical Indicators: (1) Combination with RA Disease Activity Indicators: “In our model, SIRI correlates positively with DAS28 scores (r=0.42, p<0.01) and synovial blood flow on joint ultrasound. We recommend combined use: a patient with DAS28>5.1 and SIRI>1.326 indicates coexisting high disease activity and mortality risk, thus requiring intensified immunosuppression and cardiovascular protection.” (2) Incorporation into Cardiovascular Risk Stratification Systems: “The integration of our model’s results with the Framingham Risk Score (for example, high - risk group with Framingham score≥20% and nomogram total score≥150) can identify RA - specific high - cardiovascular - risk populations, guiding decisions on aspirin or anticoagulant therapy.”. (3) Combination with Imaging and Biomarkers: “For high - risk patients according to the nomogram, additional tests such as coronary artery calcium (CAC) scoring and carotid ultrasound are recommended. If CAC exceeds 100 or plaques are unstable, dual antiplatelet therapy should be initiated. Continuous monitoring of IL - 6 and TNF - α levels is also advised; sustained elevation suggests uncontrolled inflammation, indicating a need to change biologics.”. 4. Clinical Application Scenarios: Primary - care workers can utilize the nomogram to rapidly score and identify high - risk RA patients (e.g., those with a total score>120) for referral, taking into account CRP, ESR, and swollen joint count to avoid missed diagnoses. Upon referral to a higher - level hospital, doctors can implement multidisciplinary management (e.g., collaborative care by rheumatology and cardiology). This involves formulating individualized treatment plans based on risk stratification (e.g., combining TNF - α inhibitors with SGLT2 inhibitors for high - risk patients) and incorporating results from echocardiography (LVEF), pulmonary function tests (DLCO), and bone density measurements (DXA). For surgical patients, preoperative assessment using this model can predict postoperative complication risks in RA patients undergoing joint replacement. High - risk surgical candidates should delay the procedure until their SIRI falls below 1.0 and should be assessed in conjunction with ASA classification, cardiopulmonary exercise testing (CPET), and troponin levels.

Strengths of this study include: (1) This study uses the NHANES database (1999 - 2018), a large - scale, long - term national health and nutrition survey with wide demographic coverage, ensuring representative and generalizable results that enhance scientific credibility. (2) NHANES provides comprehensive clinical and laboratory data, including detailed medical history, physical examination, and laboratory test results, enabling thorough analysis of mortality risk factors in RA patients. (3) Rigorous statistical analysis and variable screening identified three optimal predictors: age, heart failure, and SIRI. SIRI, reflecting the body’s inflammatory state, innovatively predicts RA - related mortality, offering a new prognosis assessment perspective. Its selection, based on extensive data analysis, ensures model scientific soundness and practicality. (4) Unlike Yin et al.’s univariate analysis exploring SIRI - mortality links without considering confounding indicators, this study performed LASSO and multivariate logistic regression, eliminating interference from other indicators and enhancing result convincingness. (5) The prediction model shows high performance in training and validation cohorts (AUC: 0.852 and 0.904). Calibration plots confirm consistency between predictions and observations, and DCA reveals significant net benefits of the nomogram in both cohorts. This indicates the model’s strong discriminative ability and clinical applicability, allowing doctors to quickly and accurately assess RA patients’ mortality risk. (6) As a visual prediction tool (nomogram), the model simplifies medical procedures, promotes early detection and treatment, and enables doctors to rapidly estimate RA - related death risk through score calculation, improving patient management.

The limitations of this study are as follows. First, the NHANES database, though rich in data, is retrospective and may have collection and recording biases. For example, incomplete medical histories or missing lab results in some patients can affect the model’s accuracy and reliability. Second, specific data are lacking. The NHANES database, despite being extensive, may miss detailed RA - patient - specific data like full treatment histories, medication use, and disease activity indicators, which are important for a comprehensive mortality - risk assessment. Third, the model’s external validation is lacking. Although it performs well in training and validation cohorts, it hasn’t been externally validated in different regions and medical settings, limiting its broad application. Finally, dynamic changes are not fully considered. The model, based on static data, may not fully capture the impact of RA patients’ dynamic condition and inflammatory status on mortality risk. Future research should improve data collection, expand validation, and include more potential predictors to enhance the model’s accuracy and universality. Also, more prospective, multicenter randomized controlled studies are needed to further confirm the reliability of the conclusions.

## Conclusions

This study developed a nomogram model to predict RA patient mortality using the NHANES database (1999 - 2018). The model includes age, heart failure, and SIRI as optimal predictors, showing good discrimination and clinical utility. A higher SIRI is associated with a significant drop in RA patients’ survival rate, underlining its value as an inflammatory marker for RA prognosis. The model provides clinicians with a simple, effective tool for quickly and accurately identifying high - risk RA patients, facilitating early intervention and personalized treatment, and enhancing patient outcomes.

## Data Availability

The original contributions presented in the study are included in the article/supplementary material. Further inquiries can be directed to the corresponding author.
